# Microfluidic-Driven Assembly of RNA Nanocomplexes: Design, Process Control and Translational Perspectives in Oncology

**DOI:** 10.3390/pharmaceutics18060679

**Published:** 2026-05-29

**Authors:** Ronan Pinto Nobrega dos Santos, Dana Celeste Betancourt Roldan, Muslum Guven, Lucas Campana Leite, Francisco Jacomine Madrid Furlan, Gabriel Rocha Mariano da Silva, Vitória Almeida Pessoa de Oliveira, Carolline da Silva Capriglione, Josie Pereira da Silva, José Carlos Pinto, Ismail Eş, Tiago Albertini Balbino

**Affiliations:** 1 Nanotechnology Engineering Department (PENt), Alberto Luiz Coimbra Institute for Graduate Studies and Research in Engineering (COPPE), Federal University of Rio de Janeiro (UFRJ), Rio de Janeiro 21941-594, Brazil; ronan.santos@coppe.ufrj.br (R.P.N.d.S.); francisco.furlan@coppe.ufrj.br (F.J.M.F.); gabriel.silva@coppe.ufrj.br (G.R.M.d.S.); vitoriaalmeidapessoa@ufrj.br (V.A.P.d.O.); carolline.capriglione@coppe.ufrj.br (C.d.S.C.); josie.silva@coppe.ufrj.br (J.P.d.S.); 2 Biomedical Engineering Department, Ankara University, 06830 Ankara, Türkiye; danacelesteroldan@ankara.edu.tr; 3 Turkish Energy, Nuclear and Mineral Research Agency, Boron Research Institute, 06530 Ankara, Türkiye; muslum.guven@tenmak.gov.tr; 4 Chemical Engineering Department (PEQ), Alberto Luiz Coimbra Institute for Graduate Studies and Research in Engineering (COPPE), Federal University of Rio de Janeiro (UFRJ), Rio de Janeiro 21941-594, Brazil; lleite@peq.coppe.ufrj.br (L.C.L.); pinto@peq.coppe.ufrj.br (J.C.P.); 5 Integrated Technologies Application and Research Center, Ankara University, 06790 Ankara, Türkiye

**Keywords:** microfluidic nanocomplex assembly, RNA delivery systems, lipid–polymer hybrid nanoparticles, controlled flow synthesis, tumor-on-chip, personalized cancer, process optimization

## Abstract

RNA-based therapeutics are becoming increasingly important in oncology, particularly following the rapid development of mRNA technologies during the COVID-19 pandemic, but their success strongly depends on how efficiently they can be delivered to target cells. Microfluidic technologies have redefined the design and manufacturing of RNA-based nanocomplexes, as they enable precise control over physicochemical features that are critical for clinical translation in oncology. This review examines recent developments in microfluidic-assisted synthesis of RNA nanocarriers, with a focus on cancer applications. Through a detailed analysis of material systems, device architectures, and formulation strategies, we explore how laminar flow environments enable reproducible encapsulation, tunable particle size, and improved payload stability. We examine the microfluidic assembly of lipid nanoparticles and polymeric carriers for RNA delivery, highlighting strategies to enhance durability, bioavailability, and cellular uptake. Advancements in process optimization, including flow parameter refinement and inline monitoring, are discussed alongside the influence of device geometries on mixing dynamics and nucleation. Beyond formulation, we explore the integration of microfluidics with tumor-on-chip platforms to evaluate transport, penetration, and therapeutic response in physiologically relevant cancer models. By connecting technological innovation with preclinical application, this work outlines the trajectory toward next-generation, personalized RNA nanomedicines enabled by microfluidic precision.

## 1. From Conventional Nanocarriers to Microfluidic-Engineered RNA Therapies in Personalized Oncology

**Figure 1:**
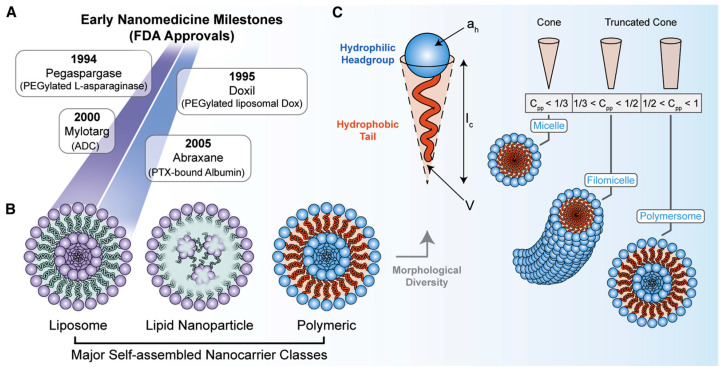
Developments in nanomedicine and schematic overview of self-assembling nanocarriers. (**A**) Timeline of early milestones in the clinical translation of anticancer nanotechnologies. (**B**) Representative categories of self-assembling nanocarriers, including liposomes, lipid nanoparticles (LNPs), and polymer-based systems. (**C**) Illustration of the critical packing parameter concept for amphiphilic polymers, highlighting how variations in hydrophobic chain volume (V), chain length (lc), and hydrophilic headgroup area (ah) influence the preferred morphology of the resulting nanostructures. Reprinted with permission from [2].

Gene therapy has been established as an important therapeutic approach for the treatment of cancer, with the capacity to modulate disease processes at the molecular level; however, its clinical translation strongly depends on the development of safe, efficient, and reproducible delivery systems. The clinical success of RNA-based therapies has evidenced the need for delivery platforms that are both biologically effective in overcoming off-target effects, immune activation, and organ specificity, and also capable of being reproducibly manufactured at scale [1]. In this context, self-assembled nanocarriers have gained prominence as a key approach, with the ability to encapsulate and protect RNA while supporting controlled delivery to target tissues. Figure 1 depicts the evolution of self-assembled nanocarriers by highlighting key milestones in the clinical translation of nanotherapeutics and the diversification of carrier architectures, from liposomes to lipid and polymer-based systems. By incorporating the critical packing parameter concept, the figure captures how molecular geometry governs nanostructure morphology, an important principle for the rational design of nanocarriers and their transition from bulk fabrication to microfluidic-controlled assembly [2].

**Figure 2:**
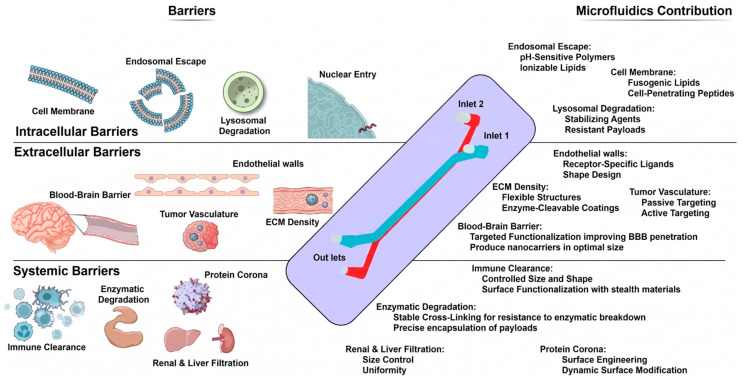
Microfluidic-driven strategies to overcome biological barriers in RNA nanocarrier delivery. The schematic shows the principal systemic, extracellular, and intracellular barriers that limit RNA nanocarrier performance and highlights how microfluidic technologies control particle size, surface properties, functionalization, and RNA encapsulation to overcome these constraints under continuous-flow conditions. Reprinted with permission from [7].

Lipid nanoparticles (LNPs) and their polymeric counterparts have long been explored for nucleic acid delivery; nonetheless, challenges such as batch variability, limited encapsulation efficiency, and poor targeting remain critical barriers to therapeutic advancement [3,4]. Efforts to address these limitations require a shift in how nanocarriers are designed and assembled, leading to increasing interest in microfluidic technologies. In comparison with earlier methods for the fabrication of nanoparticles, microfluidics distinguishes itself by precisely controlling formulation parameters at the microscale [5] and rapidly screening nanostructure formulations in an automated and continuous manner. This flexibility enables the fabrication of a wide range of nanostructures [6]. Critically, the predictable control offered by microfluidics over mixing regimes and local reaction environments results in highly uniform and reproducible nanocarrier populations, paving the way for the production of materials that meet stringent quality standards required for biomedical and clinical use [2]. Despite these advances, nanocarriers must still navigate a series of complex biological barriers that limit their therapeutic efficacy. Figure 2 depicts these challenges across intracellular, extracellular, and systemic levels, including immune clearance, enzymatic degradation, and inefficient intracellular trafficking, and outlines microfluidic-driven strategies developed to overcome them.

## 2. Microfluidic Technologies for RNA-Loaded Nanocomplex Assembly in Cancer Research

### 2.1. Microfluidics as a Platform for Controlled Nanoparticle Assembly

Microfluidics represents an engineering approach in which fluids are confined and manipulated within microscale channels, creating a controlled environment for the precise and reproducible assembly of RNA-loaded nanocomplexes. These nanocomplexes range from LNPs to polymeric and hybrid systems, including block-copolymer micelles, polymersomes, lipid–polymer hybrid nanoparticles, and nucleic acid-based assemblies. Microfluidic platforms are well suited to handle this diversity, as they allow precise control over formulation conditions across different material systems [8]. By utilizing various architectures, such as single-phase continuous flow and multiphase or droplet-based devices, these systems operate under laminar flow conditions—where fluids move in parallel streams with minimal mixing—which ensures predictable particle formation, continuous operation, and highly monodisperse product streams [9]. Within this microscale environment, operational variables such as flow rates, reagent concentrations, and temperature can be precisely modulated. Such process-level control is critical for dictating the resulting nanostructures’ physicochemical characteristics, like size distribution, morphology, surface charge, and monodispersity [3], as well as encapsulation efficiency and payload stability. These parameters are vital for the delivery of unstable RNA molecules, as they directly impact cellular uptake, endosomal escape, gene expression, and the nanocarriers’ ability to navigate biological barriers (depicted in Figure 2). Beyond the precise control of these physicochemical attributes, the clinical viability of microfluidics-based assembly is further improved through high encapsulation efficiency and reproducible production.

A significant benefit of microfluidic nanoparticle assembly is the capacity to attain high encapsulation efficiency and repeatability, which are critical for clinical translation. The conditions of rapid mixing and laminar flow minimize the degradation of RNA and promote uniform distribution of the payload within the lipid matrix. Furthermore, microfluidic platforms facilitate the integration of in-line analytical tools to monitor particle size and encapsulation in real time, thereby improving batch-to-batch consistency and accelerating formulation development [4,10]. These features directly support the transition of RNA nanocarriers from research laboratories to clinical and industrial environments by enabling consistent manufacturing and scalable production [11].

### 2.2. Assembly Mechanism

**Figure 3:**
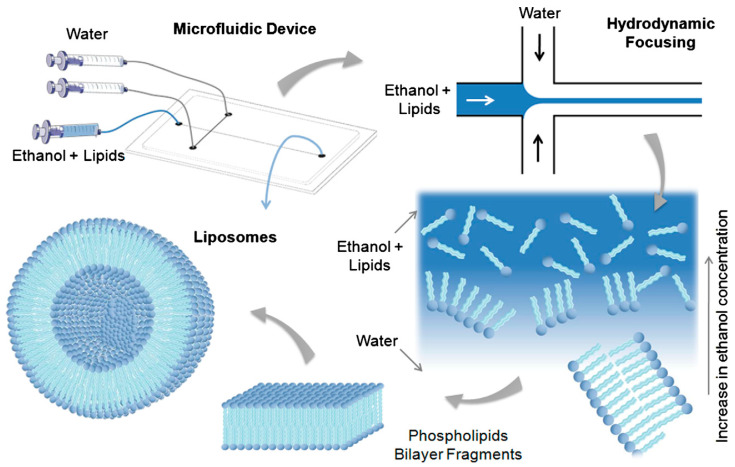
Microfluidic self-assembly process for liposome formation in a hydrodynamic focusing device. The central lipid–ethanol stream is hydrodynamically compressed by two aqueous side streams; as ethanol diffuses into water, phospholipid bilayer fragments form and close into vesicles, yielding uniform liposomes. Reprinted with permission from [15].

Microfluidic platforms utilize networks of microscale channels to manipulate the mixing of reagent solutions under laminar flow conditions. The general mechanism of nanoparticle assembly within microfluidics involves rapid and controlled mixing of organic and aqueous phases, resulting in supersaturation, nucleation, and growth of nanoparticles [12,13]. This process is primarily driven by rapid solvent exchange, where diffusion of the organic solvent into the aqueous phase reduces lipid solubility and induces spontaneous self-assembly, governed by the interplay between thermodynamic driving forces and kinetically controlled mixing conditions. The laminar conditions of microscale flow contribute to enhancing reproducibility, as mixing times are predictable and mass transfer is dominated by diffusion, not turbulence [14]. In this context, key operational parameters such as the flow rate ratio (FRR) between aqueous and organic phases and the total flow rate (TFR) critically influence nanoparticle size, size distribution, and uniformity by regulating mixing time and supersaturation kinetics. To exemplify the mechanisms underlying controlled nanoparticle formation within microfluidic environments, Figure 3 illustrates the hydrodynamic focusing approach used for liposome self-assembly. In this common configuration, a central stream containing lipids dissolved in ethanol is compressed by two converging aqueous streams, generating highly controlled interfacial mixing zones where diffusion drives the self-assembly of phospholipid bilayer fragments. As these fragments reorganize and close, uniform liposomal vesicles are formed, highlighting how microscale flow regimes govern mass transfer and vesicle size distribution, offering a demonstration of how microfluidics bridges molecular thermodynamics with process engineering in nanomedicine [15]. Moreover, microfluidic assembly offers clear potential for scalability through parallelization and modular device designs [10]; however, this aspect will be discussed in detail in a later section of this review.

### 2.3. Effects of Microfluidic Geometries and Mixing Strategies on Nanocomplex Assembly

#### 2.3.1. Passive and Active Mixing Strategies

Microfluidic mixer design is a critical determinant of LNP self-assembly, as channel geometry governs how fluids mix and thereby influences particle characteristics. In microfluidic LNP synthesis, Reynolds numbers remain low, typically in the range of Re ≈ 10–50 for gentle, lab-scale mixing, and increasing to Re ≈ 100–500 in high-throughput or high-flow microfluidic systems, thereby maintaining laminar flow conditions rather than turbulence. Although Reynolds number establishes that flow remains laminar, the Péclet number governs mass transport behavior by comparing convective and diffusive contributions. Under these conditions, high Péclet numbers indicate that convective transport dominates over molecular diffusion, such that purely diffusive mixing becomes impractical within realistic channel lengths. Consequently, mixer geometry becomes the primary lever to accelerate solvent exchange and control nanoparticle nucleation. Based on these mixing principles, microfluidic mixers are commonly classified as passive or active, depending on whether mixing is achieved through channel geometry alone or through external energy inputs [16]. Passive micromixers rely on geometrical features to enhance diffusive or chaotic advection-based mixing, whereas active mixers employ external energy fields such as pressure, thermal, electrical, magnetic, or acoustic actuation to induce convective perturbations. In the context of nanoassembly, passive micromixers are predominantly employed due to their simple design, ease of fabrication, and ability to provide stable and reproducible laminar mixing conditions without the need for external actuation. Table 1 summarizes representative microfluidic mixer geometries relevant to nanoparticle assembly. This distinction is particularly relevant for understanding the mixer geometries employed in RNA nanocomplex and LNP synthesis.

**Figure 4:**
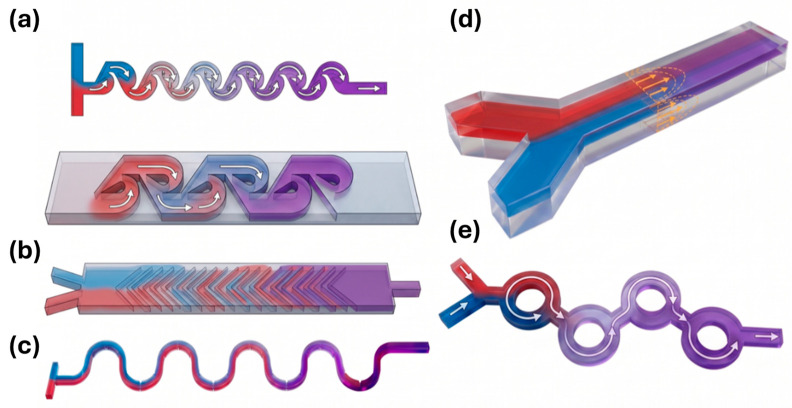
Passive micromixer geometries used as a conceptual framework for mixer classification. (**a**) Tesla micromixer. (**b**) Herringbone micromixer. (**c**) Serpentine micromixer. (**d**) Y-Junction micromixer. (**e**) SAR (split and recombine) micromixer.

**Figure 5:**
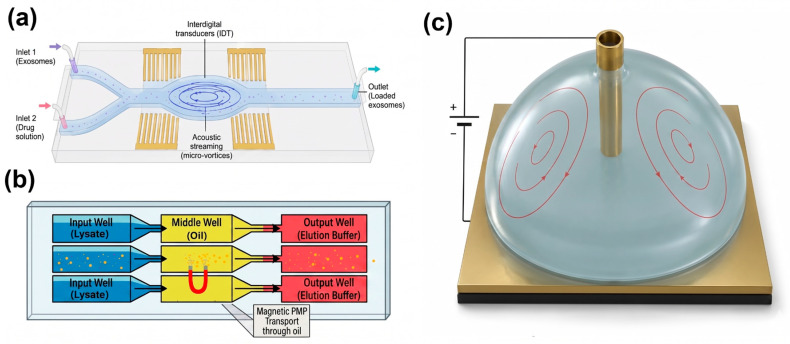
Representative active microfluidic and electrohydrodynamic platforms for enhanced mixing and biomolecular loading applications. (**a**) Schematic illustration of an acoustofluidic microfluidic platform integrating interdigital transducers (IDTs) to generate acoustic streaming and micro-vortices for active mixing and exosome/drug loading within a microchannel. (**b**) Schematic representation of an immiscible filtration assisted by surface tension (IFAST) device for rapid nucleic acid extraction and transport using magnetic particles across immiscible phases. (**c**) Schematic illustration of electrohydrodynamic mixing mechanisms in sessile droplets using a conductive electrode pin to induce internal recirculating vortices for rapid droplet mixing.

These mixing strategies are realized through a range of microfluidic geometries, each designed to manipulate fluid interfaces and enhance mass transfer under laminar flow conditions. Key design approaches include the introduction of curvature, obstacles, and three-dimensional structures to promote interfacial stretching, folding, and chaotic advection. Figure 4 and Figure 5 depict major passive and active micromixer designs, respectively. Although Figure 4 summarizes the fundamental categories of passive micromixers, more recent studies have expanded this design space through optimized Tesla-type geometries, serpentine and herringbone hybrids, obstacle-assisted layouts, and three-dimensional passive architectures that improve interfacial renewal and mixing efficiency under laminar conditions. Recent reviews also highlight that passive micromixer development has increasingly focused on balancing mixing performance with pressure drop, manufacturability, and application-specific operation ranges [25].

#### 2.3.2. Fundamental Geometries

The simplest microfluidic geometries for nanoparticle formulation are T-shaped and Y-shaped junctions, which bring two miscible streams into contact at a single intersection [16]. In these passive diffusive mixers, convective mixing is minimal; the two fluids flow side-by-side in the outlet channel, and mixing occurs primarily by molecular diffusion across the interface. The interface’s length and the residence time in the channel determine the extent of solvent exchange and thus the LNP formation. As a result, mixing performance is strongly dependent on flow rate and channel length, with the mixing length (distance to achieve ~95% mixing) growing roughly proportional to Pe in a simple T/Y channel. At high flow rates (high Pe), a very long channel is needed for diffusion to complete mixing, whereas at lower flow rates, the necessary mixing length shortens, but the overall mixing time remains long (due to slow molecular diffusion) [26]. Y-junctions can offer a slight improvement over 90° T-junctions: a wider collision angle between inlets promotes greater interfacial stretching, yielding faster mixing than a right-angle T [27]. Nevertheless, without additional mixing aids, T/Y mixers often produce larger LNPs or broad size distributions due to incomplete rapid mixing. The two streams may even flow in parallel (laminar stratification) if their momenta are low, delaying nanoprecipitation of lipids into particles. In practice, achieving sub-100 nm, monodisperse LNPs with a plain T-mixer requires either very high flow rates (to induce some inertial or weak secondary flows) or adding downstream mixing structures to perturb the flow. Lab-scale T-mixer results are often irreproducible and difficult to scale, since slight changes in flow can cause the streams to deflect instead of mixing, leading to variable nanoparticle outcomes [8]. If the inlet velocities are high enough to approach the transitional flow regime (Re ≈ 2000–4000, rarely attainable in microchannels), mixing efficiency improves, but typical microfluidic dimensions preclude fully turbulent flow [26,27].

Mathematically, the diffusion-limited mixing time in a T/Y channel can be estimated by:

(1) $$t_{mix}=w^{2}/2D$$

where w is the characteristic width of the fluid interface. For two equal-viscosity fluids, the interface width is roughly proportional to the volumetric flow ratio. If one stream (lipid-in-solvent) and the other (aqueous) flow, side by side, the steady-state width of each stream in the channel is given by their flow rate fraction (adjusted by viscosity) [28]. For example, in laminar flow, one can write:

(2) $$w_{organic}=\frac{w}{1+\alpha },\;\;w_{aqueous}=\frac{\alpha w}{1+\alpha }\;\;\;\;\;\;\;with\;\alpha =\frac{\mu_{aqueous}Q_{1}}{\mu_{organic}Q_{2}}$$

Here, Q1 and Q2 are flow rates of aqueous and organic phases and μ are their viscosities. This relation shows that increasing one stream’s flow (or viscosity) shrinks its width in the channel, thereby shortening the diffusion path. In practice, creating a very narrow interface in a T-mixer requires a high flow rate ratio (FRR) between the streams, effectively similar to hydrodynamic flow focusing. Without such focusing, the broad solvent interface in T/Y mixers can lead to undesirable intermediate ethanol fractions during mixing. Notably, if a large co-flow region exists where the local mixture is ~50% aqueous–50% solvent, the lipid self-assembly can proceed via unfavorable pathways (e.g., forming bilayer fragments or aggregates), yielding larger LNPs with lower encapsulation efficiencies. Such phenomena have been observed at low-flow or suboptimal T-mixer conditions, where LNPs ended up significantly larger and less uniformly encapsulated compared with those formed under rapid mixing [28]. Therefore, while T- and Y-geometries are useful in early development or as baseline devices, they often require either flow optimization or integration of mixing enhancers to produce nanoparticles meeting the desired size and polydispersity criteria.

Several studies utilized T-junction mixers for LNP synthesis, demonstrating variability highly dependent on formulation specifics. Jürgens et al. [29] used a simple T-junction mixer with ionizable lipid Dlin-MC3-DMA, helper lipid DSPC, cholesterol, and PEGylated lipid DMG-PEG 2000 in a molar ratio of 50:10:38.5:1.5. The organic phase (lipids in ethanol) was mixed with mRNA (coding for eGFP) in 25 mM sodium acetate buffer (pH 4) at an FRR of 3:1 (aqueous/organic). Operating at a low total flow rate (TFR) of 5 mL/h, they reported LNPs with an average hydrodynamic diameter of approximately 125 nm (DLS). The resulting mRNA encapsulation efficiency (EE) was measured at about 80%.

Further investigations varying the PEGylated lipid content demonstrated size tuning. Kumar et al. [30] formulated LNPs based on a Dlin-MC3-DMA/DSPC/cholesterol core composition (51:10:39 mol%), while systematically varying the molar fraction of the PEGylated lipid DMG-PEG, using a T-junction mixer at an FRR of 3:1. This study reported smaller LNPs sized at 58 nm (1.4–1.5 mol% PEG), 47 nm (4–5 mol% PEG), and 46 nm (6–8 mol% PEG). Encapsulation efficiencies were notably high at 96%, 81%, and 82%, respectively. Biological performance was assessed through gene silencing of Factor VII in mice and cytokine analysis in human whole blood. Kulkami et al. [31] developed another formulation, KC2/DSPC/Cholesterol/PEG (50:10:38.5:1.5 mol%), prepared via a T-junction, which yielded particles in the 20–60 nm range. Additionally, Abrams et al. [32] studied LNPs targeting mRNA liver and spleen formulated with CLinDMA/Cholesterol/PEG-DMG (50:44:6 mol%) using a T-junction, resulting in LNPs of roughly 140 nm in size with an EE of 82%.

Although the present review is focused on RNA nanocomplexes, related studies on DNA condensation highlight an additional level of complexity in diffusion-driven microfluidic assembly. Because DNA molecules are substantially longer than typical RNA cargos, their compaction may involve stochastic encapsulation of only a few DNA strands per particle, with direct consequences for particle size and size distribution. In this context, Iliescu and Tresset [33] reported a microfluidic strategy based on slow diffusion through a water stream to achieve size-controlled DNA compaction, obtaining nanoparticles with hydrodynamic diameters of approximately 30 nm. This example reinforces that nucleic acid length and diffusion conditions must be carefully considered when comparing DNA and RNA assembly regimes in microfluidic systems.

#### 2.3.3. Hydrodynamic Flow Focusing Mixers for LNP Formulation

Hydrodynamic flow focusing (HFF) mixers are designed to overcome the limitations of purely diffusive mixing by drastically reducing the diffusion distance between fluids. In a typical HFF device (also called a sheath-flow or coaxial mixer), a central fluid stream (e.g., lipid in ethanol) is injected at a relatively low flow rate, and one or two outer streams of aqueous buffer are injected at higher flow rates from side channels. The faster side (sheath) streams “sandwich” and constrict the central stream, focusing it into a thin thread. This focused stream width can be orders of magnitude smaller than the total channel width W, dramatically shortening the diffusion path for solvent exchange [6,34]. In essence, the HFF geometry trades volumetric efficiency for mixing efficiency: a large volume of sheath fluid is used to accelerate mixing of a smaller core stream [35]. The degree of focusing is controlled by the flow rate ratio (FRR), defined as the ratio of sheath (aqueous) flow to core (organic) flow. Higher FRR yields a narrower central stream (approximately in a symmetric 3-inlet design) and thus a shorter mixing time. The mixing time in an ideal 2D flow focus scales roughly with the square of the focused width; therefore, increasing FRR has a quadratic benefit in speeding up diffusion-driven mixing [13]. Notably, in the pure diffusion regime, the TFR (i.e., absolute velocity) does not change the diffusive mixing time when FRR is fixed. Instead, higher speeds simply convect the mixing zone further downstream without accelerating diffusion. This means an HFF mixer provides consistent mixing performance over a range of flow rates, as long as the geometry and FRR are maintained, which is a useful attribute for scale-up by parallelization.

HFF mixers have been widely adopted for LNP production, including in early mRNA vaccine development efforts [4]. They can reliably produce sub-100 nm particles with low polydispersity by ensuring rapid solvent dilution around nascent lipid clusters. For example, using a 3-inlet focusing chip at FRR ~3:1 (aqueous:organic) and moderate total flow (a few mL/min), one can obtain LNPs ~70–100 nm in diameter with PDI ~0.1 and high encapsulation efficiency [28]. At higher FRRs or multi-stage focusing, even smaller particles (~50 nm) are achievable, albeit at the cost of excessive dilution; Seder et al. [36] report tuning to ~50–60 nm by high dilution conditions. One drawback of flow focusing is the requirement for large volumetric ratios of buffer, which dilutes the product stream (often >75% of the output is sheath fluid). This mandates a downstream buffer exchange or concentration step (ultrafiltration), as commonly noted in LNP reviews like that of Mehta et al. [6], which discusses trade-offs in buffer ratios. Additionally, simple 2D focusing (in a planar microchannel) forces the focused stream against the channel walls, which can cause uneven mixing or lipid deposition. Innovations like 3D flow focusing (using a coaxial capillary or multilayer channel to center the core flow in all dimensions) avoid wall contact and improve mixing symmetry; HFF devices are often classified into 2D vs. 3D forms in such reviews [37]. However, 3D-focused devices are more complex to fabricate and align, posing challenges for manufacturing scale-up, as noted in instrumentation reviews such as Qin et al. [38], where fabrication complexity is addressed.

Despite these considerations, HFF geometry offers a highly controllable mixing environment; by adjusting FRR and flow rates, researchers can tune the LNP size and polydispersity in a predictable manner. In general, higher FRR (stronger focusing, hence faster mixing) yields smaller LNPs, as the rapid quenching of solvent around lipids favors quick nucleation of many small particles rather than growth of a few large ones. There is a practical upper limit to FRR beyond which gains diminish, and material waste is significant; Seder et al. [36] note diminishing returns at extreme FRR. Within optimal ranges, flow-focusing mixers strike a balance between mixing efficiency and material usage, making them a popular choice for both lab-scale formulation and even clinical manufacturing. For example, the NanoAssemblr™ Ignite system in early vaccine production employs a variation in flow focusing, as discussed in microfluidics reviews on drug delivery systems [37].

Jürgens et al. [29] investigated HFF for LNPs based on the Onpattro formulation using Dlin-MC3-DMA, DSPC, cholesterol, and DMG-PEG 2000 in a molar ratio of 50:10:38.5:1.5. The devices utilized were manufactured in-house from PDMS, featuring a channel size of 300 µm (width) × 100 µm (height) × 22 mm (length). Operating at a low TFR of 5 mL/h and an FRR of 3:1 (aqueous/organic), this study reported LNPs encapsulating scrambled siRNA or uncapped EGFP mRNA. The overall RNA recovery was remarkably high, ranging from virtually full recovery (97–100%) for mRNA LNPs to 73% encapsulation efficiency (EE) for siRNA LNPs. Biologically, the best EE attained by the HFF device yielded LNPs capable of promoting 50% gene silencing of GAPDH mRNA ex vivo in human precision-cut lung slices (PCLS). However, in vitro flow cytometry analysis showed that cellular uptake (Median Fluorescence Intensity, MFI) of LNPs formulated by HFF was significantly reduced (by half) compared with those formulated by T-junction or staggered herringbone mixers, despite their high-quality attributes.

Eş et al. [39] focused on overcoming the challenges posed by DSPE-PEG incorporation, which often leads to micelle-like aggregate formation and precipitation in diffusion-based systems, by modulating the ionic strength of the aqueous phase. Using an HFF device at an FRR of 10 and a TFR of 120.12 μL/min and fluid flow velocity at 143 mm/s, they synthesized stealth cationic liposomes (SCLs) formulated with EPC/DOTAP/DOPE/DSPE-PEG2000 (1 mol% PEG). Their system at 25 mM lipid concentration in the ethanolic dispersion, and they obtained CLs with an average size of 146.8 ± 4.9 nm, a PdI of 0.12 ± 0.04, and a ζ-potential of 55.5 ± 2.1 mV, demonstrating the high reproducibility of this method. However, despite their stability, PEG-containing formulations typically exhibit very low productivity and encapsulation yields in microfluidic systems, mainly due to reduced lipid–solute interactions and steric hindrance introduced by the PEG moieties.

The HFF principle has also been broadly applied to complex pDNA (LPXs/polyplexes), offering detailed insights into its advantages and limitations for gene therapy vectors. Balbino et al. [15] utilized a single straight channel microfluidic device (SMD) based on HFF to synthesize pDNA/cationic liposome (CL) complexes (LPXs) using EPC/DOTAP/DOPE (50:25:25 mol%). Operating at an average fluid flow velocity of 140 mm/s and an FRR of 5 (CL/pDNA), the SMD required temperature control (4 °C cooling platform) to maintain complexes with desired characteristics (≈120 nm, PDI 0.22, Zeta potential +53.6 mV). Without cooling, the particle size and PDI were significantly higher, suggesting that diffusion-based mixing alone was inefficient unless the reaction kinetics were slowed by temperature.

Comparing HFF (microfluidic mixing) versus conventional bulk mixing (BM) at high pDNA loading (molar charge ratio R ± 1.5), the microfluidic method successfully generated smaller and more homogeneous structures [40]. Structural analysis using small-angle X-ray scattering (SAXS) revealed that BM resulted in aggregates with a high number of stacked bilayers (N ~ 5), whereas the microfluidic process yielded smaller structures with a lower number of stacked bilayers (N ~ 2.5). This structural distinction led to the microfluidic-assembled lipoplexes achieving significantly higher in vitro transfection efficiencies in PC3 cancer cells at R ± 1.5 compared with the bulk method.

#### 2.3.4. Chaotic Micromixers: Herringbone Grooves and Dean Flow Designs

Passive chaotic micromixers employ engineered channel geometries (grooves, ridges, bends, spirals) to generate transverse flows, stretching and folding fluid streams, thus accelerating mixing even under laminar conditions. Unlike straight-channel mixers (T, Y, or focusing) that rely purely on diffusion, chaotic micromixers continuously perturb and recombine fluid interfaces using secondary flows (eddies, vortices), thereby progressively reducing the local diffusion distance (or “striation thickness”) with each mixing iteration [41].

One of the canonical designs is the Staggered Herringbone Mixer (SHM), first reported by Stroock et al. [41]. The device features a repeating pattern of shallow grooves on the channel floor, arranged in a herringbone motif whose orientation alternates each half-cycle. As fluid traverses each groove, it is forced into a helical motion: the top and bottom layers are split, rotated, and merged, yielding a sequence of stretching and folding events. Notably, though the flow continues to be strictly laminar (Re typically ≲ 100), the geometry induces chaotic advection (i.e., sensitive dependence on initial conditions in Lagrangian trajectories) rather than turbulence; in additional modeling work, Stroock and McGraw [42] used a lid-driven cavity analogy to reproduce the advection patterns and mixing quality as a function of geometry.

A hallmark result of SHM theory and experiment is that the required mixing length scales approximately logarithmically with the Péclet number (for Pe beyond a threshold), which implies that elevating flow velocity (hence Pe) imposes only a weak penalty on mixing length, and so mixing time can still decrease with increased throughput. Stroock et al. [41] showed that a ten-fold increase in flow speed led to only ~2.3× longer mixing length but ~4.3× shorter mixing time [42]. This contrasts with simple diffusive mixers, where mixing length and time typically scale linearly with velocity (or Pe). In practice, this weak sensitivity enables high-throughput operation without loss of mixing fidelity.

The SHM principle has been adopted in microfluidic LNP formulations. As noted in microfluidic-LNP reviews, chaotic mixers (e.g., herringbone) are among the most commonly used devices in LNP production and are employed in NanoAssemblr systems [43]. In many implementations (e.g., NanoAssemblr Benchtop), lipid and aqueous streams merge via a Y-junction and then flow through herringbone-ridge sections. The induced chaotic advection yields mixing on the order of a few milliseconds, enabling formation of uniform LNPs (typically 50–100 nm, PDI ~0.1) with high encapsulation efficiencies (often >90%) [43]. SHMs provide enhanced control over LNP assembly compared with simpler microfluidic geometries, primarily by promoting efficient chaotic advection under laminar flow, which improves batch-to-batch reproducibility and consistency in biological performance. However, this mixing strategy intrinsically limits per-channel throughput, as increasing Reynolds numbers beyond the laminar regime compromises mixing predictability and nanoparticle uniformity. To overcome this limitation, Shepherd et al. [44] implemented a parallelized microfluidic device (PMD) comprising 128 SHM channels with uniform flow distribution, enabling a >100-fold increase in production rate while preserving nanoparticle quality. The PMD consistently generated small LNPs (<85–90 nm) with narrow size distributions and maintained biological efficacy, including significant in vitro gene silencing and >90% Factor VII knockdown in vivo, demonstrating that SHM parallelization effectively decouples mixing quality from throughput constraints.

Beyond herringbone geometries, Dean-flow/spiral micromixers harness curvature-induced secondary flows to enhance mixing. In curved or spiral channels, centrifugal forces cause faster fluid to shift toward the outer wall and slower fluid toward the inner wall, establishing counter-rotating vortices (Dean vortices). The strength of this effect is quantified by the Dean number,

(3) $$De=Re\;\sqrt{\frac{Dh}{2R}}$$

where *Re* is the Reynolds number, *Dh* is the hydraulic diameter, and *R* is the channel bend radius. As *De* increases, mixing is enhanced: for low *De*, flow remains nearly plug-like; for intermediate *De*, stable Dean vortices form, enabling strong transverse mixing; and for high *De*, mixing may approach more turbulent-like behavior in microchannels [45,46]. In the context of microfluidic LNP assembly, these Dean-flow-induced secondary motions are particularly advantageous, as they dramatically accelerate solvent exchange between the lipid-containing organic phase and the aqueous phase, thereby governing nucleation kinetics, particle growth, and final size distribution. Spiral designs exploit this by routing fluid through multiple loops, thus repeatedly generating Dean flows and extending mixing distance without enormous channel lengths [47]. By simulation and experiments, it has been determined that the dominant mixing mechanism in spiral micromixers is chaotic advection driven by Dean flows in rectangular cross-sections [48].

Another class of chaotic mixers uses baffles or sharp bends in planar channels to create local recirculation zones. Each baffle induces eddies and stream folding, reducing diffusion path length. However, baffles increase hydraulic resistance and may cause particle deposition or clogging under high concentration or viscosity; so careful geometric optimization is necessary. For example, a recent S-shaped baffle micromixer study Bie et al. [49] showed that asymmetric vortices enhance mixing while minimizing pressure penalty. In general, both Dean-flow designs and baffle mixers fall into the split-and-recombine category, where flows repeatedly divide and merge, akin to multilamination. Design guidelines (number of loops, baffle angle, curvature radius) have been derived from computational and experimental studies to optimize mixing at targeted Re/Pe regimes.

Overall, chaotic micromixers (herringbone, spiral, baffle) achieve mixing times in the range of 1–10 ms in microfluidic channels [50]. This rapid mixing arrests the growth phase of LNP assembly, favoring formation of smaller particles. Shear rates generated by secondary flows further accelerate solvent diffusion and may suppress aggregation. Critically, to date, no evidence suggests that these shear conditions damage mRNA payloads: LNPs formed by chaotic microfluidic mixing retain high biological activity, transfection efficiency in vitro and in vivo.

These physical principles have been directly exploited in microfluidic platforms for RNA-loaded LNP synthesis, where chaotic mixing enables rapid and reproducible formulation screening and size control. Chen et al. [51] utilized a chaotic mixer (CM, implying SHM) device for high-throughput screening of ionizable lipids (lipidoids). This approach rapidly generated highly effective siRNA LNPs with diameters ranging from 60 to 90 nm, depending on the TFR, achieving encapsulation efficiencies around 80%. Further formulation studied by Belliveau et al. [52], and they showed LNPs based on DLinKC2-DMA/DSPC/Cholesterol/PEG-c-DMA (40:11.5:47.5 − x:1 + x mol%) could be generated, ranging from 28 to 54 nm with EE > 95%. Eş et al. [39] demonstrated high-throughput synthesis of conventional and stealth cationic liposomes (CLs/SCLs) using a chaotic advection-based microfluidic device (CA-MD) at a high TFR of 500 μL/min. Unlike the diffusion-driven D-MD, the CA-MD successfully synthesized SCLs (EPC/DOTAP/DOPE/DSPE-PEG(2000) 1 mol%) without observable aggregation. At an FRR of 1, the conventional CLs and SCLs both had sizes of around 187 nm and high homogeneity (PDI < 0.15). This CA-MD process increased productivity by over 70 times compared with the D-MD process, demonstrating significant scale-up potential. SAXS and Cryo-TEM confirmed both conventional CLs and SCLs exhibited unilamellar structures.

Lin et al. [53] designed a sophisticated 3D-printed Omnidirectional Sheath-flow Enabled Microfluidics (OSEM) device combining a 4-way hydrodynamic flow focusing region upstream with a SHM downstream. This device utilized durable methacrylate-based photopolymers that support high flow rates. The use of a 4-way sheath flow was found critical for generating a uniformly focused, circular center flow, which enabled the production of LNPs with low polydispersity, even at high flow rates. They employed the Moderna lipid formulation (ionizable lipid C12-200 based) to encapsulate SARS-CoV-2 spike protein mRNA at a constant N/P ratio of 6. This system demonstrated production at a high throughput of 60 mL/min, which is 5-fold higher than commercially available multi-thousand-dollar micromixers. This study reported LNPs with an average hydrodynamic diameter of 85.68 ± 0.81 nm, very low polydispersity (6.42% ± 0.59%), a near-neutral zeta potential of −3.78 ± 1.00 mV, and an impressive mRNA encapsulation efficiency of 93.28% ± 8.28%. They found that LNP size decreased with increasing TFR (from 1 to 6 mL/min), enhancing encapsulation efficiency, a result consistent with faster mixing kinetics preventing intermediate disk-like lipid structures formation.

Jung et al. [54] introduced a microfluidic system featuring five repeated donut ring structures. The formulation used DOTAP (ionizable lipid), DSPC, DSPE-PEG(2000), and β-Cholesterol in a 34.5:40:0.5:25 molar ratio, with EGFP mRNA mixed in PBS buffer at an N/P molar ratio of 3:1. Operating at a TFR of 18 mL/min, an FRR of 1:4, and LNPs sized at 105.5 ± 1.56 nm with a low PDI of 0.11, encapsulation efficiency was measured at approximately 80%. The biological performance was robust, showing approximately 72.3% EGFP expression in MDA-MB-231 cells, proving highly comparable to the Lipofectamine 3000 control; the system was capable of continuous and monodisperse LNP production in less than 2 min.

#### 2.3.5. Active Micromixers for LNP Production

Active micromixers compete against purely geometric (passive) designs by injecting external energy (e.g., acoustic, electric, magnetic, thermal, or pressure oscillations) into otherwise laminar microflows. Active perturbations can (i) stabilize long-duration continuous runs by mitigating fouling, (ii) expand the operable throughput window without sacrificing nanoscale size control, and (iii) introduce on-demand tunability of mixing intensity via actuator set-points (voltage, frequency, power). By promoting convective disturbances, secondary vortices, or rapid interfacial deformation, they can shorten the mixing time, narrow size distributions, and potentially raise encapsulation efficiency during lipid–aqueous solvent exchange. Their relevance to LNP formation has been emphasized in recent reviews on microfluidic LNP technologies and acoustofluidics, which note that nucleation and growth are highly sensitive to the local mixing rate and spatiotemporal uniformity [4].

Bolze et al. [55] established a continuous ultrasound-assisted antisolvent precipitation scheme for LNPs in a multilamination micro-mixer. In-process ultrasonication at frequencies in the range of 250–100 kHz suppressed fouling and enabled uninterrupted operation for 4–7 h, whereas the same chip clogged within minutes without ultrasonication. Typical products exhibited mean diameters of ~80 ± 2 nm (DLS) with polydispersity reported for standard runs; the study also documented operation at TFR on the order of tens of mL min^−1^ (e.g., solvent 6, antisolvent 30, sheath 20 mL min^−1^), highlighting the feasibility of higher-throughput active operation. They argue that the main ultrasound benefit in their configuration is continuous in situ cleaning (cavitation-assisted) that preserves stable mixing. While this study did not involve testing with mRNA or nucleic acids, later work by Nag et al. [56] pointed out the risk of mRNA degradation as a consequence of sonication at high frequencies (50 kHz and above), which was tested for periods of 100 s.

Modarres and Tabrizian [57] developed an Electrohydrodynamic (EHD)-driven micromixer in which an applied AC electric field induces interfacial instabilities and cross-stream motion, enabling highly monodisperse nanoscale liposomes by nanoprecipitation. The device allows for the tuning of liposome size and dispersity by controlling the electric-field parameters together with FRR and TFR, illustrating a field-addressable route to regulate self-assembly kinetics beyond what passive geometries afford.

Despite advances, major barriers remain for pharmaceutical implementation of active micromixers: added device complexity and cost, actuator integration and reliability under GMP constraints, and the need to validate payload integrity (e.g., mRNA) under acoustic/electric fields at scale. State-of-the-art reviews of microfluidic LNP manufacturing underscore these opportunities and gaps and call for hybrid architectures in which passive geometries are complemented by intermittent active perturbation to combine robustness with controllability. Where passive mixers reach limits (e.g., fouling at high solids loadings, sensitivity to upstream disturbances), ultrasound-assisted operation offers an immediately translatable lever to maintain product quality over long runs; EHD mixing is attractive when fine, real-time control over size/polydispersity is prioritized, provided electrical actuation is compatible with the formulation and regulatory constraints. Moving forward, quantitative targets for active LNP modules include ≥ mL min^−1^ throughput with PDI < 0.1 and EE > 90%, under actuator powers that demonstrate payload safety and batch-to-batch reproducibility [4].

#### 2.3.6. Comparative Performance of Microfluidic Geometries

Each microfluidic geometry imposes a characteristic mixing profile that influences key quality attributes of mRNA LNPs; notably particle size, size distribution (PDI), and encapsulation efficiency (EE). Table 2 collects representative performance outcomes reported for various mixer types. Broadly speaking, fast, chaotic mixing (e.g., SHMs, spirals, high-FRR focusing) tends to yield smaller, more monodisperse LNPs with high encapsulation, whereas slow diffusive mixing (T- or Y-mixers or manual bulk mixing) often produces larger and more heterogeneous particles.

For example, manual pipette mixing of lipid and mRNA frequently yields LNPs in the ~130–150 nm range with PDI > 0.2 [58]. In contrast, microfluidic impinging-jet or vortex mixers have been shown to reliably produce ~90 nm LNPs with PDI ~ 0.1 and enhanced transfection potency (often >4× higher gene delivery compared with bulk mix). Staggered herringbone micromixers have been reported to generate LNPs of ~50–80 nm (commonly ~80 nm in vaccine settings) with PDI ~0.05–0.15 and encapsulation efficiencies of ~90–95% [41]. Baffle and Dean-flow mixers (e.g., toroidal ring designs) generally yield ~90–110 nm LNPs for mRNA applications. In one example, a ring micromixer (NxGen-style) produced ~100 nm plasmid-DNA LNPs with ~80% EE; for mRNA, similar designs often exceed 90% EE after further optimization [28]. Droplet-based microfluidics, though less thoroughly documented in the context of mRNA LNPs, have demonstrated generation of ~100 nm or smaller nanoparticles with very narrow distributions, thanks to the uniform mixing each droplet imposes. In some works, droplet-fusion methods achieved PDI < 0.1 and high encapsulation efficiencies [59].

In conclusion, mixer geometry strongly influences mRNA-LNP assembly by controlling the balance of convective mixing, diffusion, and shear. Chaotic mixers (herringbone, spiral, baffle) consistently produce smaller, more monodisperse LNPs with high EE. Simpler intersecting designs rely more on diffusion and often yield larger, more polydisperse particles unless aided by high flow or additional mixing features. Ongoing developments in computational fluid dynamics and microfluidic design continue to improve predictive capability, and recent experimental systems (2022–2025) indicate feasible directions for more robust and scalable mRNA-LNP manufacturing.

## 3. Advances in Microfluidic-Fabricated Nanocomplexes

### 3.1. LNPs for RNA Delivery via Microfluidics

The development of LNPs as carriers for RNA delivery has marked a pivotal advance in nanomedicine, particularly with the advent of microfluidic technologies that enable their precise and reproducible assembly. The origins of LNPs can be traced to the evolution of liposomal drug delivery systems in the late 20th century, but it was the integration of microfluidic methods in the 2000s that truly revolutionized their formulation for nucleic acid therapeutics. Early landmark studies, such as those by Jahn et al. [60], demonstrated that microfluidic hydrodynamic flow focusing could produce LNPs with narrow size distributions and high encapsulation efficiencies, setting a new standard for the field.

**Figure 6:**
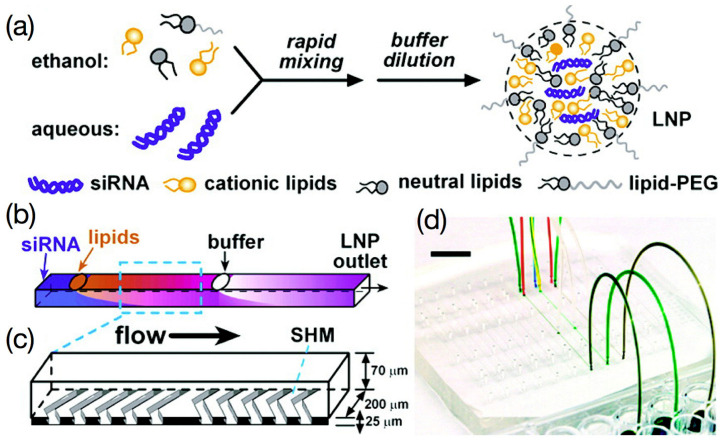
Schematic representation of LNP formation and device architecture for siRNA encapsulation. (**a**) Rapid mixing of ethanol (lipid phase) and aqueous (RNA phase) streams drives the spontaneous self-assembly and aggregation of lipids into LNPs. (**b**,**c**) Microchannel and staggered herringbone micromixer structures enhance interfacial contact and mixing efficiency. (**d**) Photograph of a parallel microfluidic chip with 24 channels for large-scale LNP synthesis. Adapted and reprinted with permission from the American Chemical Society [61].

To illustrate the underlying principles of microfluidic lipid-RNA assembly, Figure 6 schematically depicts the formation mechanism of siRNA-loaded LNPs through rapid mixing of ethanol and aqueous phases. In this process, lipid molecules in the organic phase spontaneously self-assemble around RNA strands as diffusion and dilution of ethanol promote vesicle closure and stabilization. Microchannel and chaotic micromixer configurations intensify mixing and ensure uniform nanoparticle formation, as well as the parallelized microfluidic array that enables high-throughput and reproducible LNP production. Together, these design features demonstrate controlled nanoscale aggregation and the transition from molecular components to functional delivery systems.

To provide a comparative overview of recent advances in the field, Table 3 summarizes key studies on the microfluidic assembly of LNPs for RNA delivery. The table highlights the diversity of LNP compositions, RNA cargo types, and microfluidic device designs that have been explored, as well as the main findings and innovations reported in each work. Collectively, these studies demonstrate how microfluidic technologies have enabled precise control over LNP size, encapsulation efficiency, and functional performance, supporting the rapid development and optimization of RNA-based therapeutics for a range of biomedical applications.

One of the most significant technical challenges in the field remains the precise control of LNP size and size distribution, as these parameters critically influence biodistribution, cellular uptake, and therapeutic efficacy. LNPs produced via microfluidic platforms typically exhibit sub-100 nm hydrodynamic diameters, often in the range of 50–100 nm, with narrow polydispersity, depending on formulation parameters, solvent system, and flow conditions [8]. In contrast, traditional top-down methods such as ultrasonication and high-pressure homogenization often result in broader size distributions and lower encapsulation efficiencies, underscoring the advantages of microfluidic approaches for nucleic acid delivery.

Importantly, microfluidic approaches have also facilitated the exploration of novel LNP compositions and architectures. By enabling precise control over the assembly process, microfluidics allows for the systematic investigation of how lipid composition, PEGylation, and helper lipid ratios affect RNA encapsulation, stability, and delivery efficiency. This has led to the rational design of LNPs optimized for different types of RNA (e.g., mRNA, siRNA, saRNA) and for targeting diverse tissues, including extrahepatic sites that have traditionally been challenging for nucleic acid delivery [4,8].

### 3.2. Polymeric Nanoparticles for RNA Encapsulation via Microfluidics

Polymeric nanoparticles have become foundational in the development of advanced RNA delivery systems, offering unique advantages in terms of tunable composition, biodegradability, and versatility for gene therapy. Early work focused on PLGA, chitosan, and PEG-based copolymers, which provide favorable safety profiles and customizable release kinetics for siRNA, miRNA, and mRNA delivery [69,70]. The evolution of microfluidic synthesis has enabled the precise control of polymeric nanoparticle formation through nanoprecipitation, which typically involves the rapid mixing of polymer solutions with RNA-containing aqueous phases under laminar flow conditions [5]. This technique allows for the adjustment of critical parameters, such as FRR, polymer concentration, and solvent composition, that govern self-assembly, particle size, and RNA loading [37]. The integration of targeting ligands, imaging agents, and responsive moieties is also facilitated by microfluidic platforms, supporting the development of multifunctional nanocarriers for combinatorial and personalized medicine [8].

To provide a comparative overview of recent advances, Table 4 summarizes representative studies on microfluidic-synthesized polymeric nanoparticles for RNA encapsulation. The table highlights key materials, device designs, RNA cargo types, and principal outcomes, illustrating the diversity and innovation in this rapidly evolving field.

It is observed in recent literature that, despite the remarkable diversity of polymeric materials and microfluidic strategies employed, there is still a lack of standardization in reporting key performance metrics such as encapsulation efficiency, release kinetics, and in vivo transfection outcomes [9]. While multiple studies highlight improvements in particle size control and batch reproducibility, direct comparisons are often complicated by differences in experimental design, RNA cargo, and analytical methods [80]. Furthermore, the literature reveals that most recent advances focus on optimizing physicochemical properties and modularity, but relatively few address the long-term stability, immunogenicity, or scalability of these systems for clinical translation [81,82]. This underscores the need for more systematic benchmarking and head-to-head studies to truly assess the translational potential of microfluidic-synthesized polymeric RNA nanocarriers [9].

Building on this critical perspective, it becomes clear that the field is still exploring the full potential of polymer combinations and surface modifications to address specific delivery challenges. For example, the use of composite and hybrid nanoparticles (such as PLGA cores coated with chitosan or PEG) has shown promise in enhancing cellular uptake, protecting RNA from enzymatic degradation, and enabling more precise control over release kinetics [83,84]. Composite nanoparticles based on PLGA and chitosan demonstrate enhanced transfection efficiency and sustained release properties compared with individual polymers alone, with the positive charge on the surface facilitating improved cellular uptake [84]. However, the translation of these findings into clinically relevant systems will require not only further optimization of material properties but also a deeper understanding of how these design choices impact biological interactions, immune responses, and long-term safety in vivo.

Device engineering plays a critical role in optimizing polymeric nanoparticle characteristics. Innovations such as 3D hydrodynamic flow focusing, intricate micromixer geometries, and controlled droplet generation have enabled the precise modulation of particle size, RNA loading, and surface charge, attributes that strongly influence cellular uptake, immune evasion, and biodistribution [85,86]. Three-dimensional hydrodynamic focusing can provide more homogeneous flow profiles and concentration uniformity in both horizontal and vertical planes, preventing nanoparticle deposition to channel walls, which could result in device clogging and improving size uniformity [85]. Microfluidic-based one-step methods enable efficient encapsulation of siRNA and controlled nanoparticle sizing by varying flow conditions, with the resulting size-controlled nanoparticles demonstrating minimal in vitro cytotoxicity at high RNA dosages [87]. These design advancements are central to developing next-generation delivery systems capable of bypassing biological barriers and achieving efficient gene silencing or mRNA translation in target cells and tissues.

Responsive and multifunctional systems represent another transformative direction in the field. Microfluidic techniques have facilitated the integration of stimuli-responsive polymers (e.g., pH or redox-sensitive linkers) and targeting ligands on the nanoparticle surface, supporting smart delivery strategies for precise RNA release in the tumor microenvironment or specific subcellular compartments. pH-responsive polymeric nanocarriers capable of accepting or donating protons in pathological pH conditions allow for moderate conformational changes and are mostly designed using polymers with charge-shifting capabilities [88]. The ability to co-encapsulate multiple therapeutics, including drugs and nucleic acids, in a single formulation opens possibilities for synergistic treatments, combination therapies, and personalized medicine approaches [89].

Recent literature highlights the importance of process optimization and real-time monitoring in microfluidic synthesis. In this context, in-line analytical tools and feedback control systems can support the rapid identification of process deviations and the adjustment of operating conditions during nanoparticle production, thereby reinforcing quality-by-design strategies in pharmaceutical development. These capabilities are particularly relevant for continuous and modular manufacturing schemes, in which process stability, data integration, and operational decision-making become as important as formulation performance itself [90]. Collectively, such developments are helping move polymeric RNA nanocarrier production toward more automated and analytically informed manufacturing workflows.

### 3.3. Hybrid Nanoparticles for RNA Delivery via Microfluidics

**Figure 7:**
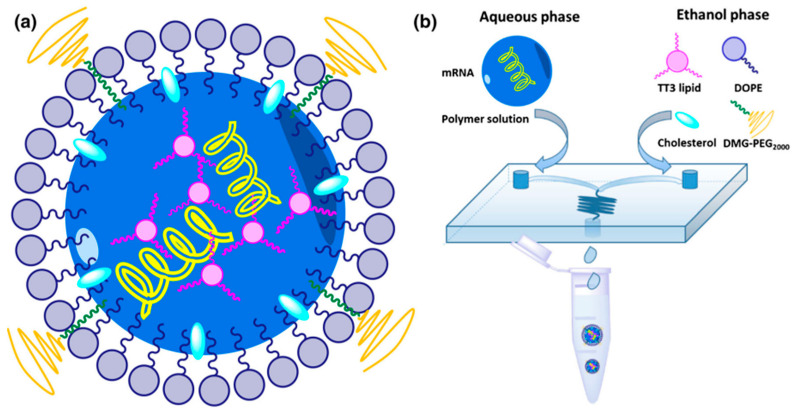
Lipid–polymer hybrid nanoparticle (LPN) structure for mRNA delivery. (**a**) Schematic architecture showing a biocompatible polymeric core containing the mRNA cargo, enveloped by an amphiphilic phospholipid monolayer and externally coated with PEG chains for stealth and stability. (**b**) Schematic illustration of the microfluidic co-assembly process used to fabricate lipid–polymer hybrid nanoparticles, in which an aqueous phase containing the polymer–RNA complex and an organic phase containing lipid components converge under laminar flow conditions, promoting controlled solvent exchange and self-assembly into hybrid nanostructures. Adapted from [91].

Hybrid nanoparticles, which integrate lipidic, polymeric, and occasionally inorganic elements, have emerged as adaptable carriers for RNA therapeutics because they combine complementary material functions within a single architecture. Unlike single-material systems, hybrid platforms benefit from the distinct contributions of each component: lipids facilitate membrane interaction and endosomal escape, polymers improve structural robustness and release behavior, and inorganic elements may add imaging functionality or stimulus responsiveness. Within this context, microfluidic assembly is especially useful because it supports coordinated co-precipitation and interfacial organization of multiple components during nanoparticle formation, which is essential for preserving structural hierarchy in these multicomponent systems. Figure 7 schematically illustrates both the structural architecture of lipid–polymer hybrid nanoparticles and the microfluidic co-assembly process commonly employed for their fabrication.

Recent microfluidic approaches have demonstrated how compositional modularity can be exploited to engineer hybrid nanostructures. Liu et al. [5] utilized a flow-focusing geometry to co-assemble lipid–polymer nanoparticles with adjustable surface charge and size, achieving improved mRNA delivery compared with traditional formulations. Similarly, Maeki et al. [4] employed modular microfluidic platforms to fabricate lipid–polymer hybrids for genome editing, demonstrating the ability to systematically optimize FRR and material proportions to maximize encapsulation efficiency and minimize polydispersity. These studies underscore the capacity of microfluidics to customize hybrid nanocarriers for emerging modalities such as CRISPR/Cas9.

Beyond lipid–polymer hybrids, the integration of stimuli-responsive components has broadened the functional scope of these systems. Sanjay et al. [92] developed pH-responsive hybrid microcapsules using glass capillary microfluidics, illustrating how environmental triggers can be harnessed for site-specific release. Advances like this highlight the growing convergence between advanced materials and microfluidic precision to produce smart carriers capable of dynamic behavior in vivo.

Microfluidic platforms have also enabled innovative fabrication strategies for more complex structural configurations. Khan et al. [93] introduced a two-step process to create a polymer-in-lipid “Trojan” nanoparticle-in-microparticle system by using elongational-flow micromixers and subsequent encapsulation via coaxial droplet generation. This architecture facilitated independent tuning of the core and shell, allowing precise manipulation of drug release kinetics. Zhang et al. [3] emphasized the role of droplet microfluidics and flow-focusing in generating monodisperse hybrid particles with controlled morphology, reinforcing the scalability of these methods for biomedical manufacturing.

Comparative analyses have demonstrated that hybrid nanoparticles frequently outperform their single-component counterparts in RNA protection and delivery efficiency. Toudeshkchouei et al. [94] highlighted how lipid–polymer hybrids fabricated in continuous-flow microfluidic devices achieved higher stability and reproducibility, especially under conditions relevant to mRNA vaccination. However, challenges in large-scale production, long-term storage stability, and regulatory standardization have been consistently reported across the literature, underscoring the need for further optimization of device design and formulation parameters to enable clinical translation [44,95].

## 4. Innovative Process Optimization Methods in Microfluidic Nanocomplex Assembly

Process optimization in microfluidic nanocomplex assembly has become an important area of development in drug and gene delivery. The ability to precisely manipulate process parameters such as FRR, TFR, solvent composition, and mixing geometry has enabled great control over nanoparticle size, uniformity, and encapsulation efficiency. For instance, Han et al. [96] demonstrated that systematic adjustment of the FRR in hydrodynamic flow focusing devices leads to the formation of smaller and more uniform LNPs, highlighting the critical role of flow dynamics in dictating nanocomplex properties. Similarly, Javid-Naderi et al. [77] emphasized that both TFR and FRR are pivotal in governing nucleation and growth during nanoprecipitation, allowing for the fine-tuning of nanoparticle characteristics to meet specific therapeutic requirements.

The integration of advanced computational approaches, particularly machine learning and surrogate modeling, has further expanded process optimization in microfluidic systems. Saeed et al. [10] introduced modular microfluidic platforms that leverage multi-objective optimization algorithms (based on deep neural networks, random forests, and decision trees) to predict and adjust synthesis parameters in real time. This approach both enhances control over nanoparticle size and polydispersity and also supports the development of automated and high-throughput manufacturing workflows. The use of data-driven models enables rapid screening of formulation variables and device geometries, significantly reducing experimental workload and accelerating the development of optimized drug delivery systems.

Sensor integration and real-time feedback control represent another significant development in microfluidic process optimization. Alavi et al. [97] developed microfluidic devices embedded with optical and electrical sensors capable of continuously monitoring flow rates, particle formation, and compositional changes within the microchannels. These real-time data streams feed into closed-loop control systems that autonomously adjust operational parameters, ensuring consistent product quality and minimizing batch-to-batch variability. Such innovations are particularly valuable for clinical-grade nanoparticle production due to their contribution towards reproducibility and regulatory compliance.

Beyond flow and feedback control, recent studies have explored the impact of microchannel geometry and mixing strategies on nanoparticle synthesis. Shepherd et al. [8] and Saeed et al. [10] reported that the incorporation of herringbone structures, serpentine channels, and acoustic micromixing elements can improve mixing efficiency, reduce aggregation, and improve encapsulation outcomes. For example, Zhao et al. [12] demonstrated that acoustic-enhanced hydrodynamic focusing devices with periodic sharp edges can reduce mixing times to less than three milliseconds, resulting in more uniform LNPs with lower aggregation rates. These design innovations not only improve the quality of the final product but also support the scalability of microfluidic synthesis for industrial applications.

Real-time analytical techniques have also been integrated into microfluidic platforms to monitor nanocomplex assembly kinetics and product quality. Taiedinejad et al. [98] developed a microfluidic antisolvent precipitation device with access for in situ dynamic light scattering during LNP formation. As a result, they could determine particle size during nanoprecipitation for the first time within the microfluidic channel. Hauss et al. [99] combined confocal fluorescence microscopy, confocal Raman microscopy, and FRET microscopy to monitor the in situ self-assembly of liposomes within a serpentine micromixer in real time. This supports quality control during the assembly process and micromixer design for scalable nanocarrier production.

Collectively, these advances highlight the growing relevance of process optimization methods in microfluidic nanocomplex assembly. By combining hydrodynamic control, computational modeling, sensor integration, and device design, these approaches can improve formulation consistency and support more scalable manufacturing strategies. Taken together, they help address persistent challenges in nanoparticle synthesis and contribute to the broader translation of microfluidic technologies into preclinical and industrial settings as summarized in Table 5.

## 5. Implementation of Microfluidic-Driven Nanocomplexes and Outlook on Cancer Research Technologies

### 5.1. Microfluidics in Pre-Clinical Cancer Research Using Tumor-on-Chip Models

Microfluidic innovation has extended beyond nanocomplex synthesis to reshape how therapeutic efficacy is evaluated in pre-clinical oncology. While earlier sections emphasized microfluidics as a manufacturing platform for RNA-loaded nanocarriers, the application of microfluidic systems in cancer research has further grown to encompass drug screening through tumor-on-chip (ToC) devices [100,101]. Microengineered platforms can recapitulate the tumor microenvironment (TME) under physiologically relevant conditions, enabling mechanistic evaluation of delivery, transport, and response, which is critical to validating the therapeutic potential of microfluidic-assembled nanoparticles.

Unlike conventional 2D cultures or static organoid systems, ToC models leverage controlled perfusion, gradient generation, and three-dimensional architecture to replicate the biochemical and mechanical cues of native tumors. Within microchannels, cancer cells can be co-cultured with stromal elements such as cancer-associated fibroblasts, immune cells, and endothelial networks, allowing for a dynamic representation of cellular interactions [100]. Such microphysiological precision mirrors the same laminar flow principles used in nanocarrier assembly, reinforcing microfluidics as a unifying paradigm that bridges formulation and functional testing.

One of the defining contributions of ToC platforms lies in modeling the complexity of the TME, particularly the interplay between hypoxia, extracellular matrix composition, and invasive phenotypes [102]. By integrating hydrogels or ECM-mimetic scaffolds, these devices support the formation of tumor spheroids or infiltrative architectures, enabling the study of proliferation, migration, and therapeutic resistance [103]. This biomimicry is especially relevant for RNA therapies, whose intracellular delivery and endosomal escape mechanisms can now be examined in environments that mimic patient-specific tumor barriers [104].

**Figure 8:**
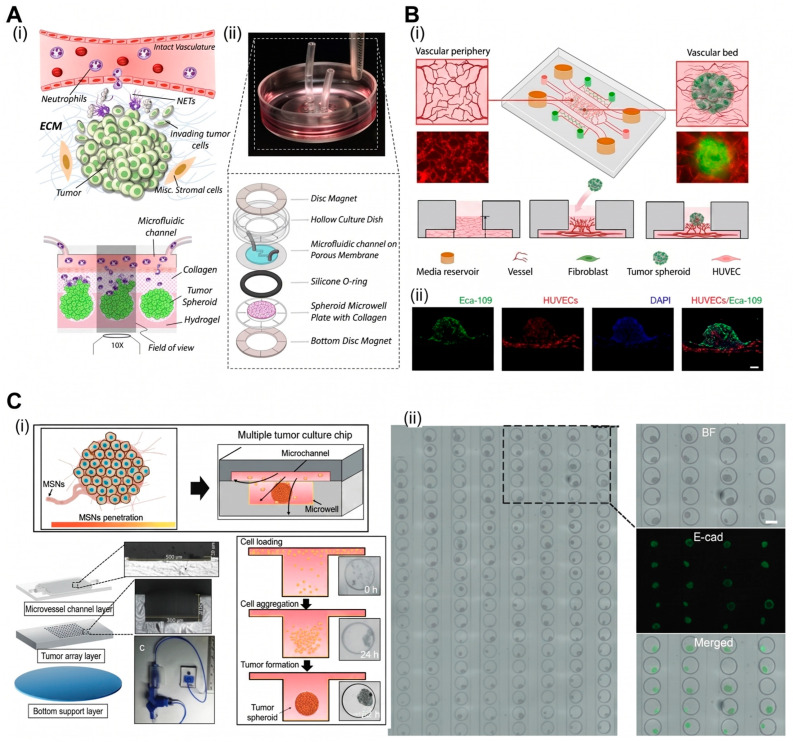
ToC platforms demonstrating the integration of microfluidic engineering and three-dimensional tumor biology for mechanistic evaluation of therapeutic transport and response. (**A**) Immune-interactive ToC model illustrating tumor–stroma interactions and neutrophil infiltration within a collagen-based hydrogel, supported by a magnetically assembled microfluidic device. (**B**) Vascularized ToC showing perfusable endothelial networks (HUVECs, red) surrounding tumor spheroids (Eca-109, green), enabling simulation of vascular–tumor interfaces and transendothelial nanoparticle transport under dynamic flow. (**C**) High-throughput microwell-based tumor culture chip allowing parallel spheroid formation and quantitative assessment of nanoparticle (Mesoporous silica nanoparticles, MSNs) penetration across multiple tumor microenvironments (reprinted with permission from [106]).

Practical implementations of ToC systems have demonstrated the feasibility of culturing cellular spheroids and vascularized tissue constructs within integrated microfluidic networks [105]. Different approaches depicted in Figure 8 illustrate the multiscale integration of microscale device design with cellular assembly, encompassing strategies such as (i) spatial organization of tumor spheroids within microwells to model tumor–stromal interactions and neutrophil infiltration dynamics; (ii) parallel cultivation of endothelial and cancer cell populations within perfusable channel architectures to recapitulate vascular-tumor interfaces; and (iii) screening platforms that enable high-throughput evaluation of particle penetration across spheroid arrays under controlled diffusion conditions. Each configuration contributes distinct mechanistic insights, from microenvironment-dependent cell behavior to transport kinetics in three-dimensional tissue-equivalent structures, thereby validating the relevance of microfluidic-synthesized nanoparticles within complex biological systems. Together, these systems exemplify how microfluidic innovation bridges nanoscale formulation and functional testing by reproducing tumor microenvironmental cues, necessary for evaluating nanocarrier efficacy and RNA-based therapeutics under physiologically relevant conditions.

Vascularized ToC models represent a major step toward physiological relevance. Perfusable microvessels formed by endothelial cells allow for the simulation of drug delivery through circulation rather than direct exposure, thus capturing the pharmacokinetic challenges faced by nanocarriers in vivo. For RNA-loaded LNPs or hybrid carriers, these systems permit analysis of transendothelial transport, vascular permeability, and intratumoral distribution. Such evaluations are essential to understanding how microfluidic-designed carriers behave within biological constraints they were engineered to overcome [107,108].

The application of ToC models to drug screening has shown improved predictive relevance compared with traditional assays. Studies involving chemotherapies such as paclitaxel, doxorubicin, or cisplatin have shown differential responses on-chip, reflecting realistic gradients and penetration limits [109]. Additionally, platforms incorporating patient-derived organoids have been used to test personalized therapeutic regimens, accurately predicting clinical outcomes in small cohorts [86]. This convergence of microfluidic fabrication and pre-clinical modeling supports a more integrated development framework, where nanoparticle synthesis, screening, and optimization occur within integrated platforms.

ToC platforms also enable mechanistic investigation of metastasis and immuno-oncology. Microfluidic systems structured to mimic intravasation and extravasation have elucidated how cancer cells navigate confined spaces and respond to shear forces during dissemination [86,107]. Likewise, co-culture models with T cells or macrophages permit real-time evaluation of immune infiltration and cytotoxicity, providing a unique testing ground for RNA-based immunomodulatory therapies [110]. These capabilities are particularly relevant as nanocomplexes increasingly incorporate targeting ligands or immune-activating payloads.

Despite their promise, ToC technologies face challenges in long-term culture stability, standardization, and clinical integration. The complexity that grants physiological relevance also demands rigorous control over oxygenation, nutrient delivery, and multi-cellular balance [108]. Future directions point toward integrating sensing technologies, AI-driven image analysis, and automated fluidic control to enhance reproducibility and scalability. As microfluidics continues to evolve from a manufacturing method into a multiscale biological platform, ToC systems may become indispensable for closing the translational gap between nanocarrier design and precision oncology [111].

### 5.2. Scalability Considerations

The capability of microfluidic assembly methods to control mixing time, shear rate, and flow patterns makes them particularly relevant in small-batch production and formulation-screening contexts, including personalized medicine applications. To move beyond these small-scale applications, process intensification through “macro-microreactor” architectures has been proposed. While demonstrated for industrial chemical processes by Zhao et al. [112], the integration of helical-shaped internals into these larger reactors provides a scalable blueprint for RNA-LNP production, maintaining mixing efficiencies that rival micro-scale devices while significantly enhancing volumetric output. The precision of such refinements is further bolstered by droplet-based microreactor platforms. By enabling high-throughput fluorescence-based screening of individual particles, this methodology allows for the precise characterization of surface acidity and pKa values—attributes that are fundamentally critical for the endosomal escape of RNA-loaded nanocomplexes in tumor cells [113]. In this setting, modular microfluidic units may support the preparation of patient-specific mRNA-LNP formulations with lower material use and improved capacity for iterative formulation refinement. Several commercial platforms (e.g., Precision Nanosystems’ NanoAssemblr series, NanoFlex, Brammer/Patheon) have packaged SHM or ring micromixer cartridges in sterilizable, disposable formats [114]. Nonetheless, microfluidic methods must translate to larger, regulated manufacturing contexts. A significant milestone in this transition is the development of one-step continuous flow synthesis systems capable of high-throughput production without compromising product stability. For instance, Yu et al. [115] reported a high-throughput microreactor system for synthesizing titanium dioxide (TiO2) nanofluids at a rate of 2 L/h, maintaining a narrow size distribution (average 28 nm) and long-term stability exceeding 40 days, which is essential for large-scale thermal management and pharmaceutical applications.

A key challenge in this regard is maintaining consistent critical quality attributes, particle size, PDI, and EE, when moving from lab-scale (µg–mg of mRNA) to clinical-scale (g–kg). The regulation of these attributes, particularly during solvent-antisolvent precipitation, is highly dependent on the reactor’s internal geometry. Studies on scale-up microreactors featuring ellipsoidal baffle mixers have shown that optimizing the baffle configuration and flow conditions allows for the precise tuning of nanoparticle diameters and distributions for insoluble bioactive compounds like apigenin, ensuring that the therapeutic profile remains consistent during the transition to mass production [112]. Turbulent mixing approaches (e.g., confined impinging jets, CIJ) have been proposed as macro-scale analogs to microfluidic chaotic mixers. For instance, Subraveti et al. [60] used a CIJ mixer to produce ~90 nm LNPs with >95% EE, comparable to microfluidic mixing performance. In their work, the CIJ method scaled mixing volumes while preserving nanoparticle performance. Additionally, the intensification of liquid-liquid mass transfer is a critical factor; systematic investigations into T-shaped macro-microreactors have utilized CFD simulations and experimental visualizations to demonstrate that mass transfer is heavily influenced by solvent selection and feed flow ratios [116]. Understanding these interfacial dynamics is vital for maintaining the uniformity of nanocomplexes when scaling up reactors to industrial capacities.

To increase the capacity for microfluidic-driven assembly, Zhang et al. [3] and Saeed et al. [10] highlighted the benefits of operating multiple microchannels in parallel, which allows for the simultaneous production of uniform nanocomplexes at higher throughput without compromising quality. This approach is particularly advantageous for scaling up nanoparticle synthesis to meet the demands of preclinical and clinical studies, as well as for the commercial manufacturing of nanomedicines.

### 5.3. Perspective Towards the Next Generation of Microfluidic-Driven Nanocomplex for Personalized Cancer Therapy

**Figure 9:**
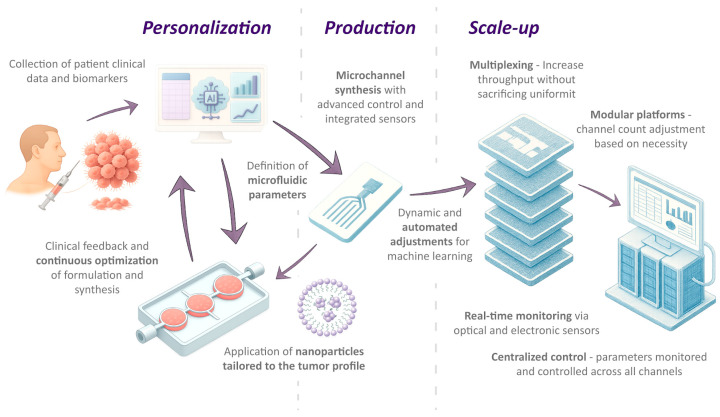
A visual perspective for the future of microfluidic-driven nanocomplexes for personalized cancer therapy. Illustrating the sequential integration of AI-based patient stratification, multi-omic cancer data analysis, and bioinformatic selection; followed by microfluidic synthesis of mRNA nanocomplexes with real-time quality control and automated ToC preclinical validation; and culminating in scalable, GMP-ready biofabrication modules for clinical translation. Capturing each modular step from data input to therapeutic production in the personalized nanomedicine workflow.

Microfluidic platforms are increasingly being explored in personalized cancer therapy frameworks because they can connect formulation development with data-guided process adaptation. In the context of RNA delivery, their relevance lies not only in nanoparticle fabrication itself, but also in the possibility of integrating synthesis parameters, quality attributes, and downstream biological testing within a more coordinated workflow [3,8,12]. This broader perspective is visually summarized in Figure 9, which outlines an integrated pathway combining patient-specific data analysis, microfluidic synthesis, real-time quality assessment, and downstream preclinical validation.

Beyond the physical aspects of nanoparticle synthesis, the integration of real-time monitoring and computational tools has expanded the analytical dimension of microfluidic development workflows [10,97]. In particular, machine learning approaches and dynamic feedback strategies may help interpret manufacturing datasets, identify parameter–response relationships, and support iterative adjustment of fabrication conditions in situ. Although still emerging, these approaches may reduce empirical optimization and improve the alignment between formulation design and biologically relevant targets [10].

Advancements in device architecture, including serpentine channels, acoustic mixers, and multilayered microstructures, have further refined particle formulation processes by improving local mixing behavior and reducing instabilities related to particle aggregation [12,44]. Rather than representing merely incremental engineering improvements, these configurations broaden the range of attainable flow regimes and formulation environments, which is important for comparing assembly pathways across carrier types and for designing more application-specific manufacturing strategies [3].

Parallel to these engineering developments, the application of ToC platforms represents a critical step toward more physiologically informative preclinical models. By recapitulating dynamic tumor microenvironments, these systems can support the evaluation of transport, penetration, cellular interaction, and treatment response under conditions that more closely resemble human physiology [117]. Importantly, their use also reinforces the need to examine how microfluidic-fabricated nanocomplexes interact with biological systems beyond efficacy alone, including issues related to heterogeneity, off-target behavior, and response variability.

Despite these advances, significant translational challenges remain, particularly with respect to the comprehensive evaluation of nanomaterial safety, long-term biocompatibility, immunogenicity, and clinically meaningful performance metrics [71,118]. Addressing these barriers will require more predictive in vivo models, stronger correlations between physicochemical properties and biological outcomes, and monitoring strategies capable of capturing complex interactions over time.

Against this backdrop of both technological opportunity and translational constraints, the convergence of microfluidics, advanced computational design, and precision nanomedicine heralds a new paradigm in personalized cancer therapy [119]. The integration of multi-omics data, automated on-chip synthesis, and high-throughput functional screening promises to deliver next-generation therapeutics that are efficacious and specifically tailored to individual patients’ tumor biology.

A forward-looking aspect of this emerging field is the incorporation of artificial intelligence and machine learning into microfluidic development workflows. These tools may support the analysis of high-dimensional omics data, the interpretation of patient-specific biological signatures, and the handling of manufacturing feedback generated during nanoparticle synthesis [10,97,120]. In the longer term, AI-enabled platforms may help predict formulation–response relationships, prioritize drug–nanocarrier combinations, and reduce the extent of empirical testing required during early development [120,121]. They may also contribute to quality control and data harmonization in regulatory settings, although their impact will depend on data quality, model interpretability, and validation under clinically relevant conditions [122,123].

Taken together, the integration of microfluidic platforms, computational stratification tools, and multi-omic data processing indicates a gradual convergence between formulation engineering and personalized therapeutic design. This emerging framework links bioinformatic analysis, controlled nanoparticle production, real-time quality assessment, and biologically relevant preclinical validation within a modular development pathway. Recent progress in ToC testing and scalable biofabrication under GMP-oriented conditions further supports the relevance of this direction, although important technical and regulatory barriers remain.

## 6. Conclusions

This review highlights how microfluidic methods have reshaped the study of RNA nanocomplex assembly by enabling closer examination of how formulation composition, mixing conditions, and device design interact during particle formation. Across lipidic, polymeric, and hybrid systems, the literature points to clear progress in engineering more sophisticated RNA carriers, but also reveals persistent gaps in standardization, comparative evaluation, and translational validation.

From a translational perspective, the most relevant advances are those that connect nanoparticle production with process analytics, biologically informative testing platforms, and scalable manufacturing strategies. At the same time, several challenges remain unresolved, including harmonized reporting metrics, long-term safety assessment, and the need for stronger links between physicochemical optimization and in vivo relevance. Additionally, limitations related to long-term stability, large-scale manufacturing robustness, and regulatory standardization remain insufficiently resolved. Continued efforts focused on process validation, comparative benchmarking, and integration with predictive in vitro models will be required to fully define the role of microfluidic nanocomplex assembly within industrial manufacturing and clinical development pipelines.

## Figures and Tables

**Table 1:** Representative passive and active microfluidic mixer architectures relevant to controlled nanoparticle and nanocomplex assembly under laminar flow conditions.

Category	Subtype	Typical Designs	Application	Reference
Passive micromixers	Parallel lamination	Basic T-mixer and Y-mixer; parallel lamination and hydrodynamic focusing concepts	A hybrid passive micromixer for gold nanoparticle synthesis (Y-junction)	[17]
	Serial lamination	Join–split–join, split–join, split–split–join and multiple intersecting microchannels	Passive micromixer with a split-and-recombine structure achieves high mixing indexes (96% and 90%), suitable for lab-on-a-chip applications	[18]
	Chaotic Advection with high Re	Wall-obstacle injection, channel-obstacle injection and zig-zag (baffle-type) channel	A high-throughput chaotic advection microreactor for preparation of uniform and aggregated barium sulfate nanoparticles	[19]
	Chaotic advection with moderate Re	Modified Tesla, C-, L-, and connected out-of-plane L-shapes; twisted and helical channels	Tesla valve micromixer used for chitosan nanoparticle production	[20]
	Chaotic advection with low Re	Slanted ribs, slanted grooves, staggered herringbone and electrokinetic surface patterns	Microfluidic Manufacturing of Polymeric Nanoparticles using staggered herringbone and two-phase reactor	[21]
Active micromixers	Thermal	Thermally induced micromixer with integrated micro-heaters	Sequential Micromixing-Assisted Nanoparticle Synthesis Utilizing Alternating Current Electrothermal Flow	[22]
	Electrohydrodynamic (EHD)	Electric-field-driven charge-convection micromixer	Electrohydrodynamic micromixing to synthesize nanoparticles via nanoprecipitation	[23]
	Acoustic	Surface acoustic wave (SAW) micromixer based on acoustic streaming	Acoustically actuated microfluidic mixer to synthesize Budesonide nanodrugs and DNA nanoparticles	[24]

**Table 2:** Representative performance of various microfluidic geometries for mRNA LNP formation. (LNP diameter and PDI are as reported for typical formulations; EE = encapsulation efficiency of mRNA. Where a range is given, it reflects different studies or conditions. References in brackets.).

Mixer Geometry	Mixing Mechanism	LNP		PDI	Encapsulation Efficiency (EE)	References
		Formulation	Size (nm)			
T- or Y-Junction	Laminar diffusive mixing at low Re; potential chaotic enhancement at high FRR or with structural modifications.	DLin-MC3-DMA/DSPC/Cholesterol/DMG-PEG 2000; PEG varied; KC2; CLinDMA/Cholesterol/PEG-DMG	125 nm		80%	[30]
			47–58 nm	0.2–0.3; PEG variation yielded smaller PDI	96%, 81%, 82%	[31]
			20–60 nm			[32]
			~140 nm		82%	[33]
Hydrodynamic Focusing	3D focusing enhances interface thinning; efficient diffusion-based mixing.	DLin-MC3-DMA/DSPC/Cholesterol/DMG-PEG 2000; EPC/DOTAP/DOPE/DSPE-PEG2000; EPC/DOTAP/DOPE	146.8 ± 4.9 nm	0.12 ± 0.04	High EE for SCLs	[40]
			~120 nm	0.22		[41]
			85–100 nm	low	73–100% depending on RNA type	[30]
Staggered Herringbone	Groove-induced chaotic advection; strong interface folding.	DLin-KC2-DMA/DSPC/Cholesterol/PEG-c-DMA; Cationic lipidoids/DOPE/Cholesterol/PEG	28–54 nm	<0.15	80–95%	[53]
			60–90 nm			[52]
Parallel SHM	Multiple SHMs operated simultaneously for scale-up.	C12-200/DOPE or DSPC/Cholesterol/lipid-PEG	<85 nm (Factor VII LNPs); 5-fold higher luciferase expression (Luc-mRNA LNPs)	<0.15	>90%	[45]
Chaotic Advection-Based Device	Enhanced chaotic mixing under high TFR.	EPC/DOTAP/DOPE/DSPE-PEG(2000) (1 mol% PEG)	187 nm (CLs/SCLs)	<0.15	High; not numerically stated	[40]
3D-Printed OSEM	Sheath-flow focused + SHM-based chaotic mixing	C12-200-based LNPs encapsulating SARS-CoV-2 spike mRNA	85.68 ± 0.81 nm	6.42% ± 0.59%	93.28% ± 8.28%	[54]
Donut Ring Spiral Mixer	Repeated vortex generation via ring structures	DOTAP/DSPC/DSPE-PEG(2000)/β-Cholesterol (34.5:40:0.5:25)	105.5 ± 1.56 nm	0.11	~80%	[55]

**Table 3:** Comparative summary of recent studies on microfluidic assembly of LNPs for RNA delivery. The table highlights LNP compositions, RNA cargo types, device designs, and key findings, illustrating how microfluidic technologies enable precise control over particle characteristics and support the development of advanced RNA therapeutics.

Ref.	LNP Composition and Ionizable Lipid	RNA Cargo Type	Device/Method	Findings/Innovation	Outcome
[62]	4N4T ionizable lipid, SM-102	mRNA	Microfluidic mixing chip	Higher translation efficiency, tunable size < 100 nm; superior to SM-102	Enhanced SARS-CoV-2 mRNA Vax
[63]	Branched cationic lipids (CL4F8-6)	mRNA	Microfluidic mixer	Lipid branching increases stability, fusogenicity, mRNA delivery	Rational design for gene rx
[64]	Library of ionizable lipids	mRNA (EPO)	In utero microfluidics	Prenatal hepatocyte targeting, robust protein replacement	In utero mouse delivery
[65]	Ionizable, helper, cholesterol, PEG	siRNA	Microfluidic device	38 nm average size; 20% higher encapsulation vs. vortex mixed	Narrower size distribution
[66]	DLin-MC3, Helper lipids	mRNA	Confocal spectroscopy and microfluidics	Kinetically controlled payload: ~3 mRNA/LNP, up to 80% empty LNPs	Capacity modulated by lipid:RNA
[44]	C12-200, DSPC, Cholesterol, PEG	siRNA, mRNA	Single channel microfluidics (vs. PMD, bulk)	<85 nm size, >90% knockdown Factor VII; 5× luciferase expression in vivo	Superior in vivo efficacy
[4]	Ionizable, helper, cholesterol, PEG	mRNA, siRNA	Modular and parallel microfluidics	Versatile, scalable, integrable platforms for LNP production and analysis	Supports high-throughput
[67]	Anionic lipids, Various	mRNA, protein	Microfluidic encapsulation	Anionic lipid assists loading and ion-ion interaction for proteins and RNA	For protein therapeutics
[68]	Standard LNPs	mRNA (on-chip synthesis)	Continuous microfluidic platform	Integrated mRNA synthesis, purification, LNP formation in one device	Fully on-chip vaccine prod.

**Table 4:** Recent studies on microfluidic synthesis of polymeric nanoparticles for RNA encapsulation.

Ref.	Polymer(s)	RNA Type	Microfluidic Strategy	Main Outcome
[71]	Poly(glutamic acid), PLGA	siRNA, mRNA	3D HFF, droplet	13–34 nm size, high EE, tunable release
[59]	PLGA-PEG	DNA, siRNA	HFF + micromixer	Longer circulation, improved stability
[72]	Chitosan	miRNA	Diffusive micromixer	Homogeneous particles, exchangeable cargo
[73]	PLGA/Chitosan	cytokine mRNA	HFF continuous	96% EE, effective immune modulation
[74]	PLGA-PEI	mRNA	Flow-focusing droplet	Core–shell, improved cellular uptake
[75]	PEG-PEI, PACE	mRNA/siRNA	Modular microfluidics	Tunable PEGylation, optimized release
[76]	Polysaccharide blends	siRNA	Combinatory screen	Diversity-oriented screen for cardiac therapy, potent cargo
[77]	PLGA, Chitosan	siRNA, mRNA	Herringbone channel	100% EE siRNA, ~80% EE mRNA, size 20–160 nm
[78]	PEG-PLys, P(DMAEMA)-b(PAA-co-BMA)	siRNA, pDNA	Micelle-forming platforms	Improved circulation, reduced aggregation, enhanced endosomal escape
[79]	pH-responsive copolymers	Cas9 RNP, pDNA	Amphiphile-based NP	Enhanced gene editing, pH-triggered release

**Table 5:** Process optimization in microfluidic nanoparticle assembly, highlighting optimization focus, device/platform, nanoparticle type, key innovation, and outcome/result.

Ref.	Optimization Focus	Device/Platform	Nanoparticle Type	Innovation	Outcome/Result
[96]	FRR	HFF	LNPss	Systematic adjustment of FRR	Smaller, uniform nanoparticles, greater reproducibility
[77]	TFR FRR	Microfluidic laminar mixing	Solid lipid NPs	Parametric control of nucleation/growth	Tuned size and loading
[29]	Machine learning optimization	Modular microfluidic chip	Polymers/Lipids	Surrogate modeling, closed-loop tuning	Improved size control and reduced polydispersity
[97]	Sensor integration and feedback	Embedded optical/electrical	Not specified	Real-time anomaly detection	Rapid process correction and consistency
[44]	Channel geometry, flow dynamics	HFF, herringbone structures	Lipid/polymer NPs	Micro-scale structuring for rapid mixing	Enhanced control, encapsulation, improved throughput
[31]	Acoustic micromixing	Acoustic-enhanced HFF	LNPs	Periodic sharp edge micromixers	Mixing time reduced to <3 ms; reduced aggregation
[100]	Multi-parameter precision control	Multiplexed microfluidic devices	Novel Drug Delivery Systems (NDDSs)	Uniform control of size and dose	Improved repeatability and uniformity in nanoformulations

## Data Availability

No new data were created or analyzed in this study.
